# Chemical Digestion-Assisted Proteomics Reveals the Extracellular Matrix Profile of Human Periodontal Ligament and its Alterations in Cultured Cell-Derived Extracellular Matrix

**DOI:** 10.1016/j.mcpro.2025.101460

**Published:** 2025-11-10

**Authors:** Lay Thant, Azusa Dobashi, Megumi Kitami, Hlaing Pwint Phyu, Mizuki Kobayashi, Yoshiki Ono, Yoshito Kakihara, Masaki Matsumoto, Masaru Kaku

**Affiliations:** 1 Division of Bio-prosthodontics, Faculty of Dentistry & Graduate School of Medical and Dental Sciences, Niigata University, Niigata, Japan; 2 Center for Advanced Oral Science, Faculty of Dentistry & Graduate School of Medical and Dental Sciences, Niigata University, Niigata, Japan; 3 Division of Dental Pharmacology, Faculty of Dentistry & Graduate School of Medical and Dental Sciences, Niigata University, Niigata, Japan; 4 Department of Omics and Systems Biology, Graduate School of Medical and Dental Sciences, Niigata University, Niigata, Japan

**Keywords:** proteomics, periodontal ligament, extracellular matrix, matrisome, chemical digestion, hydroxylamine

## Abstract

The extracellular matrix (ECM) plays a crucial role in tissue structure and function and serves as an integral component of diverse biological systems. However, the comprehensive characterization of ECM components is challenging because of the insoluble nature of highly cross-linked and glycosylated ECM proteins. This study introduces a chemical digestion-assisted proteomic approach to overcome challenges in ECM profiling, offering an in-depth analysis of the human periodontal ligament (PDL), a tissue critical for oral function and regeneration. Furthermore, we investigated alterations in the ECM composition of cultured PDL cells to provide insights relevant to tissue engineering applications. Our protocol combined chemical digestion and deglycosylation to enhance the identification of ECM proteins. Chemical digestion by hydroxylamine improved protein extraction efficiency by approximately two-fold compared to conventional chaotropic extraction, and deglycosylation increased the number of identified ECM proteins without substantially altering the ECM profile, offering robust quantification of ECM components. A sequential protein extraction approach revealed that proteins insoluble by conventional methods are primarily composed of highly cross-linked fibrillar collagen. Through the application of this technique to human PDL tissue, we present a comprehensive ECM profile for the first time, revealing a high collagen content (>80%) and identifying dominant non-collagenous ECM proteins, such as periostin, dermatopontin, and lumican. Our findings highlight the significant differences between the native ECM of PDL tissue and that produced by cultured PDL cells, emphasizing the importance of considering these variations in regenerative strategies. This study offers a robust tool for analyzing the ECM and its dynamics across diverse tissues and under various physiological and pathological conditions. The results enhance our understanding of periodontal tissue and will inform future approaches for periodontal tissue regeneration.

## Highlights

• Hydroxylamine enhanced ECM solubilization two-fold over conventional methods.
• Insoluble proteins of conventional methods were predominantly collagens.
• Deglycosylation enhances ECM protein detection without altering the overall profile.
• ECM of human PDL comprised >90% collagen, with periostin as a key component.
• Cultured PDLC ECM exhibited reduced collagen, while retaing the major constituent.

## Graphical Abstract

**Figure:**
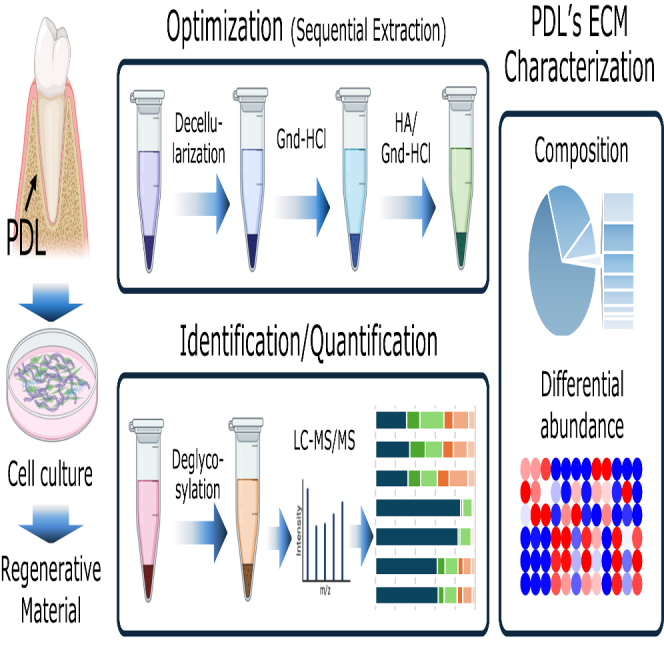

## In Brief

This study presents a chemical digestion-assisted proteomics approach to comprehensively profile the extracellular matrix (ECM) of human periodontal ligament (PDL) tissue. Findings reveal the PDL ECM is collagen-rich (>90%), with periostin, dermatopontin, lumican, decorin, and asporin as major non-collagenous components. Notable differences between native PDL tissue and cultured PDL cells highlight adaptations *in vitro*. This method overcomes traditional ECM profiling challenges, offering valuable insights for tissue regeneration and a robust tool for studying ECM dynamics across diverse biological contexts.

The extracellular matrix (ECM) is a complex network of macromolecules that determines the structure and integrity of tissues (1). The ECM provides biochemical and biophysical cues that regulate cellular processes (2); thus, the precise regulation of ECM characteristics is essential for proper tissue function and organ homeostasis (3). ECM composition and organization can substantially vary between different tissues and within the same tissue depending on the physiological state. The ECM adapts to mechanical, biochemical, and physical signals, enabling tissues to meet the demands of their functions, which highlights its significance in various biological processes, including development, regeneration, and disease progression (4). Therefore, recent research has focused on generating ECM protein atlases to elucidate the composition of diverse tissues in both physiological and pathological states (5, 6, 7, 8).

A proteomics strategy has been developed to classify data on the ECM and its associated proteins, referred to as the matrisome, which is divided into two main categories: the core matrisome (e.g., collagens, proteoglycans, and ECM glycoproteins) and matrisome-associated proteins (e.g., ECM regulators, ECM affiliated proteins, and secreted factors) (7). The matrisome database serves as a reference for analyzing the ECM composition of a diverse range of tissues and organisms (8). Despite numerous studies utilizing matrisome database for ECM quantification, protocols for accurately quantifying ECM components remain challenging. In particular, the abundance of highly cross-linked proteins such as fibrillar collagen leads to incomplete solubilization of the sample, resulting in an inaccurate protein profile (9, 10). Furthermore, post-translational modifications, such as glycosylation, should also be considered because they protect ECM proteins against proteolysis, act as physical barriers, and lead to decreased solubility (11, 12). Although the matrisome database has provided a framework for ECM analysis, its utility in capturing the dynamic and complex nature of the ECM remains challenging.

Various protein-extraction workflows have been developed to address the insolubility of the ECM (13, 14, 15, 16). These typically include a decellularization step to remove abundant and readily soluble cellular proteins, thereby improve ECM protein detection (15, 17, 18), followed by chaotropic extraction, typically using urea (UA), sodium dodecyl sulfate (SDS), or guanidine hydrochloride (Gnd-HCl) (13, 19, 20). Nonetheless, despite these efforts, the insoluble remnants remain. Some comparative studies have shown that chemical digestion either by cyanogen bromide (CNBr) or hydroxylamine hydrochloride (HA) has significant advantages for the quantification of core matrisome proteins (13, 15). HA facilitates selective hydrolysis at Asn-Gly (NG) sites under acidic conditions, enabling efficient ECM solubilization (21). Compared to CNBr, HA offers superior reproducibility, cost efficiency, and simplicity, making it a preferred method for proteomics (22). Consequently, HA has recently been the method of choice for protein extraction, focusing on the comprehensive characterization of the ECM profile (5, 13, 22, 23, 24). Moreover, we opted for Gnd-HCl as the base buffer for chemical digestion, as previous studies have demonstrated that Gnd-HCl extracts a more comprehensive range of core-matrisome proteins compared to UA or SDS (13, 25, 26). Furthermore, Gnd-HCl is fully compatible with subsequent LC-MS/MS analysis following simple desalting procedures. In contrast, buffers containing SDS necessitate extensive SDS removal, as residual SDS interferes with peptide ionization, significantly suppressing ion signals during electrospray ionization in mass spectrometry (27). Additionally, glycosylation adds significant complexity to proteins because glycosylation patterns vary in composition, structure, and branching (11). This diversity needs to be accounted for in the accurate identification and quantification of glycosylated proteins. Removal of glycans is effective in improving the detection coverage of glycoproteins and proteoglycans (13, 17, 23). Establishing a proteomic workflow with chemical digestion and deglycosylation that outperforms traditional protein extraction would enable more comprehensive and in-depth profiling of the ECM.

The periodontal ligament (PDL) is a specialized connective tissue that anchors the tooth cementum to the alveolar bone, thereby playing a crucial role in oral function. It functions as a shock absorber against masticatory forces, facilitates tooth eruption, and enables orthodontic tooth movement (28, 29). Its function and tissue homeostasis are influenced by mechanical forces and alterations in the physiological environment, resulting in modifications to the ECM profile (30, 31, 32). Furthermore, the ECM status of the PDL directly influences the mechanical properties of the tissue, particularly its tensile strength and compressive behavior (33, 34). Despite the critical importance of the PDL in oral function, its regeneration remains a substantial challenge in clinical practice (35). Among the various approaches for regeneration, the PDL cell (PDLC)-derived ECM, which is obtained through the decellularization of cultured PDLCs, is a promising candidate. The PDLC-derived ECM preserves its structural integrity and supports the repopulation of allogeneic cells (36). PDLCs cultured on the PDLC-derived ECM retain their stem cell characteristics and possess higher differentiation capability into osteoblasts, compared to those cultured on standard tissue culture polystyrene (37). Additionally, this approach stimulates the osteoblastic differentiation of allogeneic mesenchymal stem cells (38) and promotes the transformation of induced pluripotent cells into cells resembling PDL stem cells (39). Owing to these cell-instructing characteristics, the PDLC-derived ECM is considered to maintain the original ECM composition found in the native PDL tissue to some extent. However, the detailed profile of the ECM in cultured PDLCs and its differences from that in native PDL tissue remain elusive. This limitation has resulted in a substantial gap in our understanding of the role of the ECM in PDL maintenance and regeneration. Therefore, it is imperative to elucidate the unique ECM composition and structure of the PDL under the respective circumstances.

This study aimed to provide a comprehensive analysis of the ECM composition in human PDL tissue and its alterations in cultured PDLCs. Using mass-spectrometry-based proteomics, we developed an advanced analytical approach that incorporates chemical digestion and deglycosylation as sample pretreatments. This optimized workflow enables robust quantification of the ECM profile, facilitating a more thorough understanding of the complex components of the PDL. The ECM profile of the PDL provides the foundation for its structural and functional properties, which play crucial roles in maintaining the integrity of the tooth-supporting apparatus. Furthermore, insights gained from analyzing the ECM composition of cultured PDLCs will be valuable for developing novel approaches for periodontal tissue regeneration.

## Experimental Procedures

### Experimental Design and Statistical Rationale

Mouse PDLCs were isolated from the molars of 5-week-old male C57BL/6J mice (n = 8) and cultured. ECM-rich fractions were generated by decellularizing the PDLC-derived ECM (n = 9). Protein extraction was conducted using three distinct protocols, and tryptic peptides were prepared (n = 3).

Human PDL tissue was obtained by scraping one-third of the central part of the extracted premolars (n = 3). A portion of the PDL tissue underwent enzymatic digestion to obtain human PDLCs, and human PDLC-derived ECM was prepared by culturing (n = 3). Proteomic analysis was conducted using the same methodology as for the mouse PDLCs.

All raw files across all experimental groups were processed together in a single FragPipe run—comprising MSFragger searching (40), Philosopher FDR filtering (41), and IonQuant MS1 intensity-based quantification (42)—to generate a unified protein-abundance matrix. Subsequent normalization, filtering, and imputation were applied once to this matrix, and group-wise comparisons were conducted on the processed dataset. Raw MS data were searched against UniProt reference using FragPipe v23 with MSFragger v4.2 (40). Peptide and protein-level false discovery rates (FDRs) were controlled at 1% using the Philosopher package (41). Quantitative features were extracted using IonQuant v2.0 with enabling FDR-controlled match-between-runs (MBR) (42). Protein-level intensities were calculated using MaxLFQ label-free normalization (43). Matrisome proteins were selected from all the identified proteins using a Matrisome Analyzer (44) and the occupancy of each matrisome protein was calculated based on the MaxLFQ values (43) divided by the sum of the MaxLFQ values of all matrisome proteins. Matrisome profile analysis employs a comprehensive approach for data processing and statistical evaluation. The mean of three biological replicates was utilized to determine matrisome profiles. Principal component analysis (PCA) was conducted using ClustVis software (45). To identify the differentially expressed proteins, statistical significance was assessed using the Benjamini–Hochberg procedure (46). Proteins with an adjusted *p*-value (q) < 0.05 were considered statistically significant. Volcano plots display log_2_(fold change) on the x-axis and –log_10_(q) on the y-axis. The significance threshold was set at –log_10_(0.05) = 1.3. Further analysis of the data involved several bioinformatic tools. Gene-set enrichment analysis was conducted using Shiny GO 0.82 (47). Protein-protein interaction (PPI) network analysis was performed using NetworkAnalyst 3.0 (48). Correlation analyses were performed using Prism 9 (GraphPad Software, San Diego, CA, USA).

### Ethics Statement

The Niigata University Animal Experiment Ethics Committee reviewed and approved all the animal procedures (SA00532). All animal handling and experiments strictly followed the ARRIVE guidelines for animal research and reporting of *in vivo* experiments. The Ethics Review Board of Niigata University for Human Studies reviewed and approved the collection of human PDL tissue from teeth extracted after orthodontic treatment (approval number: 2023-0090). This research adheres to the ethical guidelines set forth by the World Medical Association’s Declaration of Helsinki.

### Mouse PDLC Culture

The first and second molars of each quadrant were extracted from C57BL/6J mice (male, 5 weeks old) and the PDL tissue was dissociated with Liberase DL (500 μg/ml in trypsin, Roche, Basel, Switzerland) for 60 min at 37 °C on an orbital shaker (200 rpm). Mouse PDLCs were collected using centrifugation (3000 rpm, 5 min) and maintained in alpha MEM (Invitrogen, Waltham, MA, USA) supplemented with 10% fetal bovine serum and 1% antibiotic-antimycotic solution (Sigma-Aldrich, St Louis, MO, USA). The mouse PDLCs were seeded onto a 35-mm dish (4 × 10^5^ cells/dish) and cultured for 1 week, and then another 2 weeks with ascorbic acid (50 ug/ml, Sigma) (49, 50).

### Human PDL Tissue and Cell Culture

Human PDL tissue was harvested from healthy premolars extracted due to orthodontic treatment at Niigata University Medical and Dental General Hospital. PDL tissue was scraped from one-third of the central part of the root and kept at −80 °C. Part of the PDL tissue was enzymatically digested to obtain human PDLCs in the same manner as mouse PDLCs. The human PDLCs were seeded onto a 35-mm dish (4 × 10^5^ cells/dish) and cultured for 1 week, and then another 2 weeks supplemented with ascorbic acid (50 ug/ml, Sigma) (51).

### Sample Preparation and Protein Extraction

To remove cellular proteins, cultured PDLCs and PDL tissues were treated with RIPA buffer (50 mM Tris-HCl pH8.0, 150 mM sodium chloride, 0.5% w/v sodium deoxycholate, 0.1% w/v SDS, and 1.0% w/v NP-40 substitute; Wako, Osaka, Japan) and centrifuged at 12,000 rpm for 30 min. The RIPA buffer-insoluble precipitate was referred to as the ECM-rich fraction (ECM-Fr). The ECM-Fr was thoroughly washed with PBS and kept at −80 °C until the protein extraction with the following protocols.

#### Conventional UA/SDS Extraction

The protein extraction was performed on the ECM-Fr with 60 μl of UA/SDS lysis buffer (100 mM Tris-HCl pH 8.8, 7 M urea, 2% SDS). Samples were sonicated for 30 s three times, incubated at 90 °C for 10 min, and centrifuged, and the supernatant was stored (Fr 1). The protein concentration was quantified using the Pierce BCA Protein Assay Kit (Thermo Fisher Scientific) and the sample concentration was adjusted to 0.5 μg/μl. Samples were reduced with 2.5 mM TCEP-HCl (Thermo Fisher Scientific) at 37 °C for 30 min and alkylated with 12.5 mM iodoacetamide (Sigma-Aldrich) at room temperature (RT) in the dark for 30 min. The buffer was then removed by acetone precipitation using 90% ice-cold acetone at −20 °C for 3 h and centrifuged at 15,000 *g* at 4 °C. The precipitated pellet was resuspended in 100 mM ammonium bicarbonate (ABC). Samples were finally digested with trypsin (1:100 enzyme and protein ratio) at 37 °C for 18 h, quenched by 1% final concentration of trifluoroacetic acid (TFA; Thermo Fisher Scientific).

#### Two-Step Extraction With Chemical Digestion

The protein digestion was performed on ECM-Fr with 60 μl of Gnd-HCl buffer (6 M Gnd-HCl, 100 mM ABC adjusted to pH 9.0 with NaOH). The samples were sonicated and placed on a rotator at RT for 17 h and the supernatant was stored (Fr 2). The remaining pellet was reduced and alkylated as described in the conventional UA/SDS extraction. The chemical digestion was performed in accordance with the method from previous reports, with slight modifications (5, 22). The reduced/alkylated pellet was chemically digested with 60 μl of freshly prepared HA in Gnd-HCl buffer (1 M HA, 0.2 M K_2_CO_3_ in Gnd-HCl buffer). The samples were sonicated and incubated at 45 °C on a tube rotator for 4 to 6 h until the samples were completely dissolved (Fr 3).

#### One-Step Extraction With Chemical Digestion

ECM-Fr was reduced and alkylated as described above. The samples were directly extracted with 60 μl of freshly prepared HA in Gnd-HCl buffer, sonicated, and incubated at 45 °C on a rotator for 4 to 6 h as previously described (Fr 4).

### Filter-Aided Sample Preparation (FASP)

Deglycosylation and trypsin digestion of Fr 2, 3, and 4 were performed using an FASP approach (52). Fr 2, 3, and 4 samples were loaded into the 10 kDa cutoff filter ultrafiltration units (MRCPRTO10, Merck-Millipore) and exchanged with 8 M UA in 100 mM ABC by repeated centrifugation at 14,000*g* for 45 min. The protein amount in each Fr was quantified using the Pierce BCA Protein Assay Kit (Thermo Fisher Scientific). Each Fr was divided into two groups, with or without deglycosylation, with protein adjusted to 100 μg in each sample. Deglycosylation was performed using 500 U PNGase F (New England Biolabs) in 2 M UA in 20 mM ABC on the ultrafiltration unit at 37 °C for 2 h. Following deglycosylation, the samples were desalted by thorough washing with 50 mM ABC and the ultrafiltration units were transferred to new collection tubes. The samples were finally digested with trypsin (1:100 enzyme and protein ratio) in 50 mM ABC at 37 °C for 18 h. The ultrafiltration units were then centrifuged at 10,000*g* until the solution had completely passed through the filter membrane (5–10 min), where it flowed through the trypsin-digested peptides. The trypsin digestion was quenched with a 1% final concentration of TFA.

### LC-MS/MS and Data Processing

Tryptic peptides, equivalent to 5 μg of protein, were desalted and purified using StageTips packed with polystyrene–divinylbenzene (SDB-XC; GL Sciences), then reconstituted in 20 μl of 0.1% trifluoroacetic acid. A 0.5 μg aliquot was injected (direct-injection mode) into an Eksigent NanoLC 415 system coupled to a TripleTOF 5600+ mass spectrometer (SCIEX).

Chromatographic separation was performed on an L-column2 C18 column (2 μm particles, 100 μm × 150 mm; CERI) at a flow rate of 300 nl/min with a 90-min run: 2% solvent B to 20% B over 60 min, 20% to 90% B over 5 min, held at 90% B for 5 min, and re-equilibrated at 2% B for 20 min (solvent A: 0.1% formic acid in water; solvent B: 0.1% formic acid in acetonitrile).

Data were acquired in positive-ion, data-dependent acquisition (DDA) mode. Each duty cycle (∼1.3 s) comprised one TOF MS survey scan (m/z 400–1250; accumulation time, 0.25 s; declustering potential, 80 V; collision energy, 10) followed by product-ion (MS/MS) scans of the 10 most intense precursors (high-sensitivity mode; m/z 100–1600; accumulation time, 0.10 s; declustering potential, 80 V; collision energy, 35 with a collision energy spread of 15; ion release delay, 67; ion release width, 25). Only precursors with charge states 2+–5+ were selected, and previously fragmented ions were excluded for 5 s.

Source/interface settings were: IonSpray voltage floating, 2300 V; Ion Source Gas 1, 20; Ion Source Gas 2, 0; Curtain Gas, 10; and interface heater temperature, 150 °C.

Raw.wiff files were centroid-converted to mzML using MSConvert (ProteoWizard v3.0) (53). mzML files were searched against UniProt reference proteomes downloaded in June 2025: *Mus musculus* (UP000000589; 54,791 sequences) and *Homo sapiens* (UP000005640; 83,587 sequences) with FragPipe v23/MSFragger v4.2 (40) (precursor tolerance ±10 ppm; fragment tolerance ±0.04 Da; semi-tryptic specificity allowing cleavage after lysine, arginine, and asparagine; ≤2 missed cleavages). Cysteine carbamidomethylation was set as a fixed modification. Variable modifications included methionine, proline, and lysine oxidation; N-terminal acetylation; and pyro-Glu/pyro-Cys formation. Peptide and protein-level false discovery rates (FDR) were controlled at 1% using the Philosopher toolkit with target-decoy competition (41). Quantification was performed in IonQuant v2.0 (42) (10 ppm m/z, 0.4 min RT, 0.05 1/K_0_); MBR was enabled (1 min RT, 0.05 1/K_0_; ≥3 scans, ≥2 isotopes), and MaxLFQ normalization was applied (43). In each comparison, all samples were processed in a single workflow with identical settings. Matrisome proteins were annotated with Matrisome Analyzer (44), and occupancy was calculated as each protein’s MaxLFQ intensity divided by the sum of MaxLFQ intensities for all matrisome proteins.

### Bioinformatic Data Analysis

Matrisome profile analysis employs a comprehensive approach for data processing and statistical evaluation. The mean of three biological replicates was used to determine matrisome profiles. PCA was conducted using ClustVis software (45). To identify the differentially expressed proteins, statistical significance was assessed using the Benjamini–Hochberg procedure (46). Proteins meeting the criteria of log (fold change) >|0.08| and log (FDR) > 2 were considered statistically significant and differentially expressed. Further analysis of the data involved several bioinformatic tools. Gene-set enrichment analysis was conducted using Shiny GO 0.81 (47). The number of N-glycosylated sites on each ECM glycoprotein was determined using NetNGlyc-1.0 (54). Heatmaps were generated using Morpheus (https://software.broadinstitute.org/morpheus/), and volcano plots and correlation analyses were performed using Prism 9 (GraphPad Software).

To compare mouse and human proteomes in a unified, high-confidence framework, we integrate FragPipe/MSFragger with IonQuant for label-free quantification and used the orthogene R package (54) to enable cross-species proteome comparison. First, mouse UniProt IDs were mapped to 1:1 human orthologs via orthogene. Subsequently, raw MS data from both species were searched and quantified together under identical parameters via IonQuant’s FDR-controlled MBR, as described earlier.

## Results

### Optimization of ECM Solubilization Methods (Experimental Workflow)

**Fig. 1:**
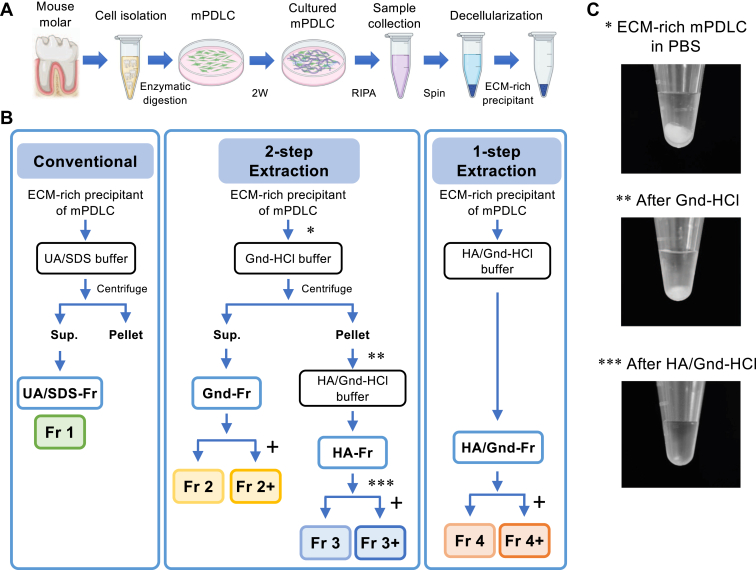
**Overview of ECM extraction methods and sample preparation.***A*, Preparation of ECM-rich samples from cultured mouse periodontal ligament cells (mPDLC). *B*, Protein extraction methods used in this study. *C*, Representative photo of the ECM-rich samples from cultured mPDLCs in PBS (∗), after Gnd-HCl extraction (∗∗), and after HA extraction (∗∗∗). ECM: extracellular matrix; UA/SDS: Urea/Sodium dodecyl sulfate; Gnd-HCl: Guanidine-hydrochloride; HA: Hydroxylamine. +: deglycosylation by PNGase F.

To determine a suitable method for ECM proteomics, particularly for protein profiling of human PDL, we prepared cultured mouse PDLCs as the starting material (Fig. 1*A*). Protein extraction from ECM-Fr was performed using three different protocols (Fig. 1*B*): conventional extraction using UA/SDS-Fr; two-step extraction with chemical digestion using Gnd-HCl buffer (Gnd-Fr), followed by HA in Gnd-HCl buffer (HA-Fr); and one-step extraction with chemical digestion using HA in Gnd-HCl buffer (HA/Gnd-Fr). Gnd-Fr, HA-Fr, and HA/Gnd-Fr were further divided into two groups, depending on whether they were deglycosylated using PNGase F. We refer to UA/SDS-Fr as Fr 1, Gnd-Fr with and without deglycosylation as Fr 2+ and Fr 2, HA-Fr with and without deglycosylation as Fr 3+ and Fr 3, and HA/Gnd-Fr with and without deglycosylation as Fr 4+ and Fr 4.

### Impact of Protein Extraction Methods on the Detection of ECM Proteins in Cultured Mouse PDLCs

**Fig. 2:**
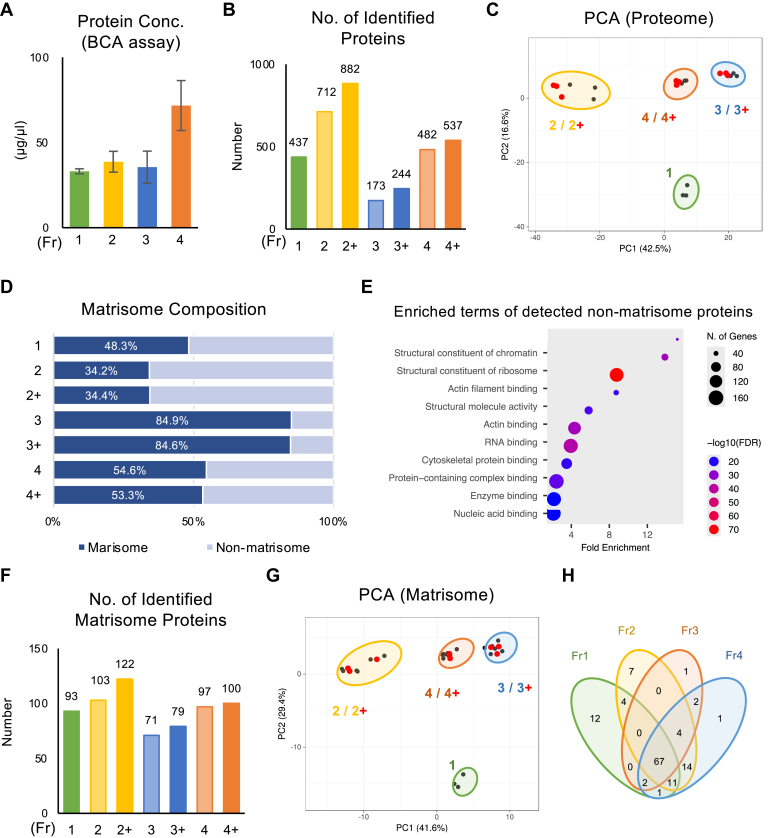
**Impact of protein extraction methods on the proteome and ECM profile of cultured mouse periodontal ligament cells.***A*, protein concentration in each fraction. *B*, number of identified proteins in each fraction. *C*, principal component analysis (PCA) of each fraction calculated based on the proteome profile. De-glycosylated samples (treated with PNGase F) are marked in red. *D*, Matrisome composition of each fraction. *E*, Gene-set enrichment analysis of detected non-matrisome proteins in Fr 4/4+. Gene ontology (molecular function) database was used for enrichment background. *F*, number of identified matrisome protein in each fraction. *G*, PCA of each fraction calculated based on the matrisome profile. De-glycosylated samples (treated with PNGase F) are marked in *red*. *H*, Venn diagram showing the overlapping of the detected matrisome protein in each fraction. ECM, extracellular matrix; Fr 1; UA/SDS-Fr, Fr 2/2+; Gnd-Fr w/o and w/deglycosylation, Fr 3/3+; HA-Fr w/o and w/deglycosylation, Fr 4/4+; HA/Gnd-Fr w/o and w/deglycosylation.

We assessed protein solubilization efficiency using visual inspection. A representative image of the sample before extraction, after Gnd-HCl extraction, and after HA/Gnd-HCl extraction using the two-step method is shown (Fig. 1*C*). Although part of the sample remained insoluble after the Gnd-HCl extraction, no visible remnants remained after the HA/Gnd-HCl extraction. The protein extraction efficiency was analyzed based on the amount of protein extracted using a BCA assay (Fig. 2*A*). Fr 1 and 2 exhibited comparable protein quantities, indicating that UA/SDS and Gnd-HCl demonstrated similar protein extraction efficiencies. Fr 3 also displayed a similar protein quantity to Fr 2, suggesting that approximately half of the protein remained insoluble. In addition to the relatively well-established two-step protocol, which involves obtaining Fr 2 followed by Fr 3, as supported by prior research (5, 22), we explored the protein extraction efficacy of a one-step protocol (Fr 4). This approach theoretically reflects the actual ECM composition of the entire sample. The protein quantity of Fr 4 was equivalent to the sum of Fr 2 and Fr 3, indicating that the one-step extraction demonstrated comparable protein extraction efficiency to that of the two-step protocol.

Although the protein extractability was comparable between Fr1 and Fr2, the number of identified proteins was greater in Fr 2 compared with Fr 1 (Fig. 2*B*). Deglycosylation using PNGase F increased the number of proteins identified in Fr 2, 3, and 4. PCA of the entire proteome showed that the different extraction methods exhibited distinct patterns, with deglycosylation partially affecting the protein profile (Fig. 2*C*). Matrisome composition analysis revealed that ∼50% of the identified proteins were matrisome proteins in Fr 1 while ∼35% in Fr 2/2+ (Fig. 2*D*). In contrast, ∼80% of the proteins in Fr 3/3+ were matrisome proteins, indicating that the insoluble remnants after Gnd-HCl extraction were primarily composed of ECM. As a consequence of the enhanced extractability of the ECM, the matrisome composition exhibited an increase in Fr 4/4+ compared to Fr 2/2+. Notably, despite decellularization being employed as a pretreatment, a negligible amount of cellular proteins remained in the ECM-Fr. The residual cellular proteins in the ECM-Fr consisted primarily of actin cytoskeleton-associated proteins (Fig. 2*E*). The number of identified matrisome proteins was the highest in Fr 2+ and lowest in Fr 3 (Fig. 2*F*). PCA of the matrisome protein profile showed that the different extraction methods exhibited distinct patterns, with deglycosylation partially affecting the protein profile (Fig. 2*G*), which was consistent with the entire proteome (Fig. 2*C*). Although the protein extraction efficiency was similar between Fr 1 and 2, these fractions demonstrated a distinct pattern (Fig. 2, *G* and *H*). Notably, Fr 2/2+ displayed large deviations between samples; however, convergence was observed for Fr 3/3+ and 4/4+.

### Impact of Protein Extraction Methods on the ECM Profile of Cultured Mouse PDLCs

**Fig. 3:**
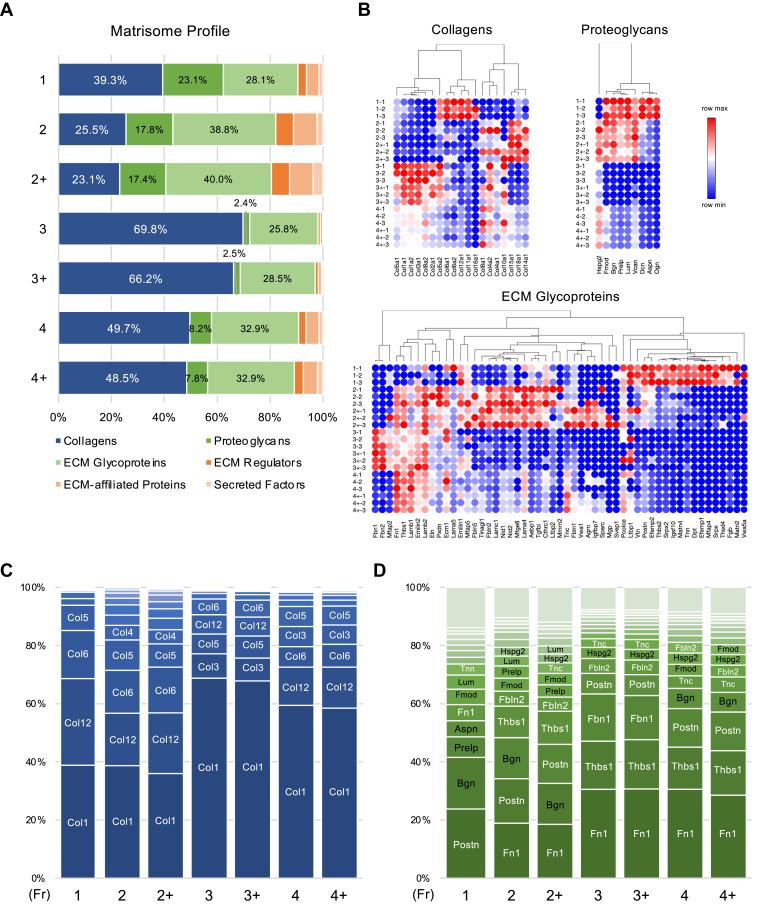
**Impact of protein extraction methods on the ECM profile of cultured mouse periodontal ligament cells.***A*, Matrisome profile of each fraction. *B*, heatmap of the core matrisome protein abundance in each fraction. Most abundant collagens (*C*) and non-collagenous ECM (*D*) in each fraction. Proteoglycans are shown in *black text*. *C* and *D*, protein order corresponds to the descending order of protein abundance in each fraction.

We analyzed the impact of extraction methods and deglycosylation on the matrisome profile (Fig. 3*A*). The matrisome profile showed that Fr 1 and 2/2+ had a similar pattern with Fr 1 possessing a higher collagen proportion, whereas Fr 3/3+, prepared from the insoluble remnants of Fr 2/2+, mainly consisted of collagen. Fr 4/4+, which most accurately represented the ECM profile of the sample in theory, displayed a higher collagen content than Fr 2/2+. Additionally, Fr 3/3+ showed lower proteoglycan proportions, representing only 2.5% of the matrisome, indicating that most proteoglycans were successively extracted using Gnd-HCl (Fr 2/2+). A heatmap showing the protein abundance of the core matrisome (i.e., collagens, proteoglycans, and ECM glycoproteins) showed a clear difference between the samples treated with or without HA (Fig. 3*B*). The abundance of collagen (e.g., COL1A1, COL1A2, COL2A1, and COL3A1) and ECM glycoproteins (e.g., FN1, FBN1, FBN2, and LAMB1) markedly increased with HA treatment (Fr 3/3+ and 4/4+). Among proteoglycans, only HSPG2 was effectively enriched by HA extraction. These results clearly demonstrated that different extraction methods, particularly with HA, affect the number and proportion of matrisome proteins.

### Impact of Extraction Methods on the Collagen Profile of Cultured Mouse PDLCs

To further elucidate the impact of the extraction methods and deglycosylation on the ECM profile, we evaluated the protein profiles of collagens (Fig. 3*C*). Type I was the most abundant collagen in all fractions. Although the protein extractability was comparable between Fr 1 and 2 (Fig. 2*A*), the proportion of collagen, notably type XII, differed in these fractions. In Fr 3/3+, approximately 70% of the collagen was type I, indicating that the insoluble protein remnants of the Gnd-HCl extract (Fr 2/2+) consisted of highly cross-linked type I collagen. In Fr 4/4+, which theoretically most accurately represents the ECM profile, type I was the predominant collagen, followed by types XII, VI, III, and V. Notably, this pattern differed markedly from that observed in other fractions. Deglycosylation did not substantially affect collagen composition, as indicated by PCA (Fig. 2*H*) and the ECM profile (Fig. 3*A*).

### Impact of Extraction Methods on the Non-Collagenous Core Matrisome Profile of Cultured MMouse PDLCs

The impact of the extraction method and deglycosylation on the profiles of non-collagenous core matrisome proteins (i.e., ECM glycoproteins and proteoglycans) was analyzed (Fig. 3*D*). Fibronectin (FN1) was the most abundant non-collagenous core matrisome protein, except with Fr 1. Although the protein extraction efficiencies were similar, the protein composition differed between Fr 1 and 2/2+, particularly on thrombospondin 1 (THBS1). In Fr 3/3+, the insoluble protein remnants of the Gnd-HCl extracts (Fr 2), abundantly contained Fibrillin-1 (FBN1), independently of other fractions.

### Impact of Deglycosylation on the Number of Detected Matrisome Proteins in Cultured Mouse PDLCs

**Fig. 4:**
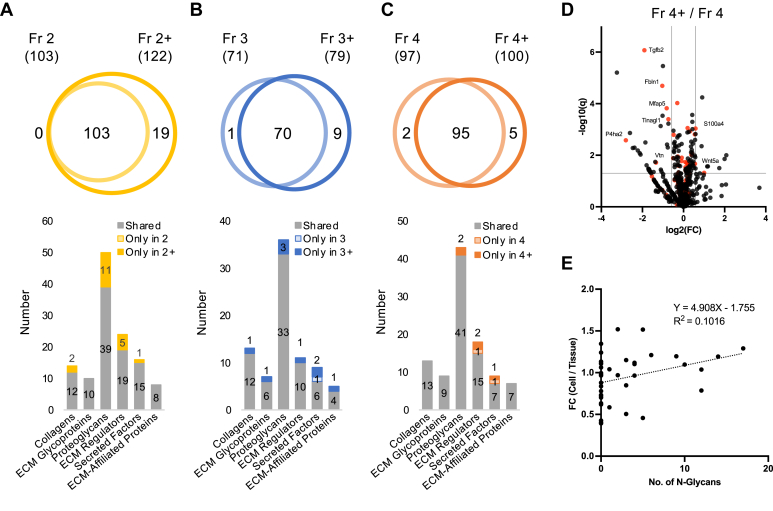
**Impact of deglycosylation on the ECM profile of cultured mouse periodontal ligament cells.** Venn diagram and bar chart showing the number of detected matrisome proteins in Fr 2 and Fr 2+ (*A*), Fr 3 and Fr 3+ (*B*), and Fr 4 and Fr 4+ (*C*). *D*, Volcano plot showing the differentially expressed proteins (DEPs) in the Fr 4+ compared to the Fr 4. Matrisome proteins are shown in *red dots*. FDR: false discovery rate. *E*, correlation analysis showing the relationship between the number of N-glycosylation site and fold change (FC) of protein abundance after deglycosylation by PNGase F on Fr 4. Fr 2/2+; Gnd-Fr w/o and w/deglycosylation, Fr 3/3+; HA-Fr w/o and w/deglycosylation, Fr 4/4+; HA/Gndgnd/HA-Fr w/o and w/deglycosylation.

While deglycosylation did not substantially affect ECM composition (Fig. 3*A*), the number of detected proteins tended to increase (Fig. 2*F*). Therefore, we analyzed the effect of PNGase F deglycosylation on the number of matrisome proteins detected in each fraction (Fig. 4, *A*–*C*). In Fr 2+, the effect of deglycosylation was evident on ECM glycoproteins and ECM regulators, whereas it expanded on other matrisome subtypes in Fr 3+. In Fr 4+, which theoretically represents the ECM profile most accurately, the effect of deglycosylation on ECM glycoproteins, and ECM regulators was observed (Fig. 4, *C* and *D*). To assess whether the impact of deglycosylation was due to the direct effect of N-glycan cleavage, a correlation analysis was conducted between the number of N-glycosylation sites and the fold change in the composition of ECM glycoproteins on Fr 4 and Fr 4+ (Fig. 4*F*). The regression analysis revealed an R-squared value of 0.1016, indicating very weak or no correlation (Fig. 4*G*). Therefore, the effect of deglycosylation cannot simply be attributed to the cleavage of N-glycans from the core protein. Although deglycosylation affects the detection of ECM proteins, the precise mechanism of action remains unclear.

### In-Depth ECM Profiling of Human PDL Tissue and its Changes in that of Cultured Human PDLCs Using Chemical Digestion-Assisted Proteomics

**Fig. 5:**
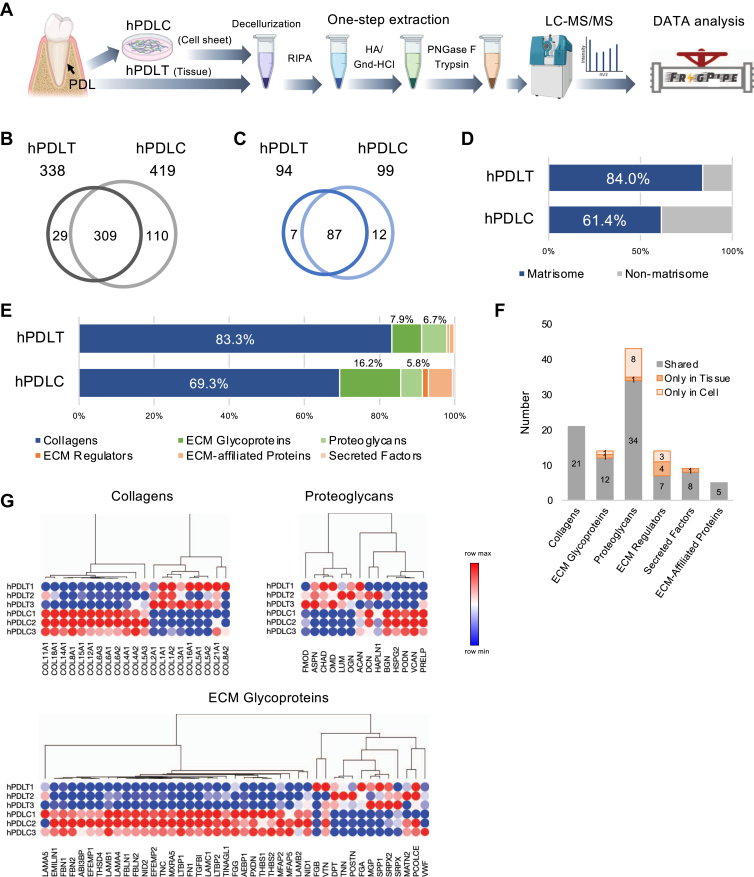
**ECM profiling of human periodontal ligament tissue (hPDLT) and cultured human periodontal ligament cells (hPDLC) using ECM proteomics.***A*, schematic of the experimental workflow. Venn diagram showing the number of detected proteins (B) and matrisome proteins (*C*) in hPDLT and hPDLC. *D*, matrisome composition of hPDLT and hPDLC. *E*, Matrisome profile of hPDLT and hPDLC. *F*, Bar chart showing the number of proteins shared by hPDLT and hPDLC within each matrisome subclass. *G*, Heatmap showing relative abundance patterns of core matrisome proteins in hPDLT and hPDLC.

We conducted an in-depth quantification of the ECM profile in human PDL tissue by employing chemical digestion-assisted proteomics. The tissue samples were solubilized using HA/Gnd-HCl (one-step extraction by chemical digestion with deglycosylation (corresponding to Fr 4+), followed by MS-based protein identification (Fig. 5*A*). We also analyzed the differences in the ECM profiles of cultured human PDLCs, a promising substrate for PDL tissue regeneration (55), compared with that of the native human PDL tissue.

After one-step chemical digestion, no visible residue remained for either human PDL tissue or PDLC-derived ECM, indicating complete solubilization at the optical level. We identified 338 proteins in the human PDL tissue, of which 94 were matrisome proteins (Fig. 5, *B* and *C*). In the cultured human PDLCs, 419 proteins were detected, of which 99 were matrisome proteins. In human PDL tissues, matrisome proteins constituted 84.0% of the entire proteome and 61.4% in cultured human PDLCs (Fig. 5*D*). The matrisome profile revealed that 83.3% of the ECM contained collagen in the human PDL tissue, whereas it decreased to 69.3% in cultured human PDLCs (Fig. 5*E*). The proportion of non-collagenous core matrisome proteins is higher in cultured human PDLCs than that in human PDL tissues. The proportion of matrisome-associated proteins (i.e., ECM regulators, ECM-affiliated proteins, and secreted factors) was also higher in human PDLCs than that in human PDL tissue, indicating accelerated ECM production under culture conditions. Among the 43 ECM glycoproteins detected, 34 were present in both tissues and cells, while the others were exclusively detected in either tissue or cells (Fig. 5*F, G*). A similar trend was observed for other matrisome subclasses, with the exception of collagens and secreted factors, which exhibited comparable patterns between tissues and cells.

**Fig. 6:**
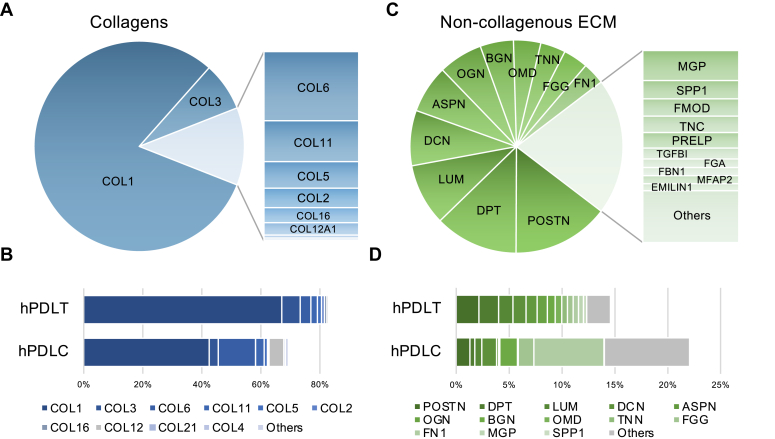
**Core matrisome profiling of human periodontal ligament tissue (hPDLT) and cultured human periodontal ligament cells (hPDLC).***A*, collagen profile of hPDLT. *B*, collagen profile comparing between hPDLT and hPDLC. *C*, Non-collagenous ECM profile of hPDLT. *D*, non-collagenous ECM profile comparing between hPDLT and hPDLC. *B* and *D*, protein order corresponds to the descending order of protein abundance in hPDLT.

Type I collagen was predominant in human PDL tissue, followed by types III, VI, and V (Fig. 6*A*). The proportion of collagen decreased in the cultured human PDLCs, with type I remaining the predominant type (Fig. 6*B*). The most abundant non-collagenous core matrisome protein in human PDL tissue was Periostin (POSTN), followed by dermatopontin (DPT), lumican (LUM), decorin (DCN), and asporin (ASPN) (Fig. 6*C*). Although the proportion of the dominant non-collagenous ECM proteins did not change significantly, many unidentified proteins in the human PDL tissue were detectable in human PDLCs (Fig. 6*D*). A complete list of the detected core matrisome proteins is presented in Table 1.

**Table 1:** Abundance of core matrisome proteins in human PDL tissue and cultured human PDLCs

Collagens					
Gene Symbol	Tissue	Cell	FC	p	
			(Cell/Tissue)		
COL1A1	40.29%	24.55%	0.44	4.7.E-04	∗
COL1A2	27.19%	18.28%	0.49	1.6.E-03	∗
COL3A1	6.27%	3.13%	0.36	2.8.E-02	∗
COL11A1	2.14%	2.91%	0.99	9.0.E-01	
COL6A2	1.26%	4.44%	2.57	3.0.E-03	∗
COL6A1	1.22%	3.50%	2.09	6.2.E-03	∗
COL6A3	1.14%	4.66%	2.99	3.0.E-03	∗
COL2A1	1.02%	0.44%	0.32	8.1.E-02	
COL16A1	0.80%	0.18%	0.16	1.3.E-01	
COL5A1	0.69%	0.56%	0.59	7.4.E-02	
COL12A1	0.64%	4.90%	5.55	1.9.E-03	∗
COL5A2	0.61%	0.43%	0.52	3.3.E-02	∗
COL21A1	0.15%	0.09%	0.41	1.1.E-01	
COL5A3	0.11%	0.14%	0.94	7.2.E-01	
COL4A2	0.07%	0.23%	2.23	9.4.E-02	
COL4A1	0.03%	0.28%	6.48	4.2.E-03	∗
COL14A1	0.02%	0.70%	28.02	1.7.E-03	∗
COL8A2	0.02%	0.01%	0.41	6.3.E-01	
COL18A1	0.01%	0.11%	5.45	4.4.E-02	∗
COL15A1	0.01%	0.08%	5.60	7.2.E-03	∗
COL8A1	0.00%	0.13%	38.31	2.3.E-05	∗

Proteoglycans					
Gene Symbol	Tissue	Cell	FC	p	
			(Cell/Tissue)		
LUM	1.36%	0.66%	0.35	3.6.E-02	∗
DCN	1.23%	1.40%	0.83	9.9.E-02	
ASPN	1.07%	0.20%	0.14	1.0.E-02	∗
OGN	0.98%	0.12%	0.09	3.9.E-02	∗
BGN	0.74%	1.68%	1.67	6.0.E-02	
OMD	0.61%	0.03%	0.04	4.0.E-02	∗
FMOD	0.27%	0.23%	0.61	1.5.E-01	
PRELP	0.25%	0.33%	0.99	9.2.E-01	
HSPG2	0.08%	0.37%	3.40	4.9.E-03	∗
VCAN	0.05%	0.74%	10.57	2.1.E-05	∗
HAPLN1	0.03%	0.03%	0.76	4.7.E-01	
ACAN	0.03%	0.03%	0.72	6.6.E-01	

ECM glycoproteins					
Gene Symbol	Tissue	Cell	FC	p	
			(Cell/Tissue)		
POSTN	2.14%	1.28%	0.44	6.5.E-02	
DPT	1.85%	0.49%	0.19	1.2.E-02	∗
FGG	0.55%	1.48%	1.95	8.6.E-02	
TNN	0.55%	0.04%	0.06	5.9.E-02	
FN1	0.50%	6.63%	9.70	6.1.E-04	∗
MGP	0.48%	0.03%	0.04	1.2.E-01	
SPP1	0.29%	0.02%	0.04	6.1.E-02	
TNC	0.26%	0.93%	2.65	2.5.E-03	∗
TGFBI	0.17%	0.57%	2.41	2.5.E-03	∗
FBN1	0.13%	0.78%	4.38	1.8.E-03	∗
MFAP2	0.13%	0.24%	1.37	1.4.E-01	
EMILIN1	0.11%	0.40%	2.65	4.6.E-03	∗
FGB	0.10%	0.05%	0.38	3.9.E-01	
NID1	0.05%	0.07%	1.08	6.2.E-01	
FBLN1	0.05%	0.39%	5.88	3.5.E-03	∗
LAMC1	0.05%	0.10%	1.54	2.6.E-02	∗
SRPX	0.04%	0.03%	0.57	2.3.E-01	
NID2	0.04%	0.08%	1.60	2.0.E-03	∗
SRPX2	0.04%	0.00%	0.09	8.0.E-03	∗
VTN	0.03%	0.04%	0.89	8.0.E-01	
AEBP1	0.03%	0.10%	2.21	5.2.E-03	∗
FBLN2	0.03%	0.48%	12.76	1.1.E-03	∗
LAMB1	0.02%	0.05%	1.53	3.5.E-02	∗
LAMA4	0.02%	0.07%	2.33	1.2.E-02	∗
LAMB2	0.02%	0.03%	1.19	3.9.E-01	
PCOLCE	0.02%	0.01%	0.53	2.3.E-01	
THBS1	0.02%	1.22%	56.18	2.8.E-02	∗
VWF	0.01%	0.02%	0.81	8.4.E-01	
MATN2	0.01%	0.01%	0.81	8.1.E-01	
LAMA5	0.01%	0.01%	1.20	8.2.E-01	
TINAGL1	0.01%	0.06%	5.37	3.0.E-02	∗
LTBP2	0.01%	0.07%	7.30	2.0.E-02	∗
EFEMP2	0.00%	0.07%	10.82	1.9.E-03	∗
MXRA5	0.00%	0.06%	20.19	2.0.E-03	∗

Proteins within each matrisome subclass are listed in descending order of MaxLFQ value in the human PDL tissue ECM. Statistically significant differences are indicated by ∗.

FC, fold change.

### Comparison of ECM Profile Between Human PDLCs and Mouse PDLCs

**Fig. 7:**
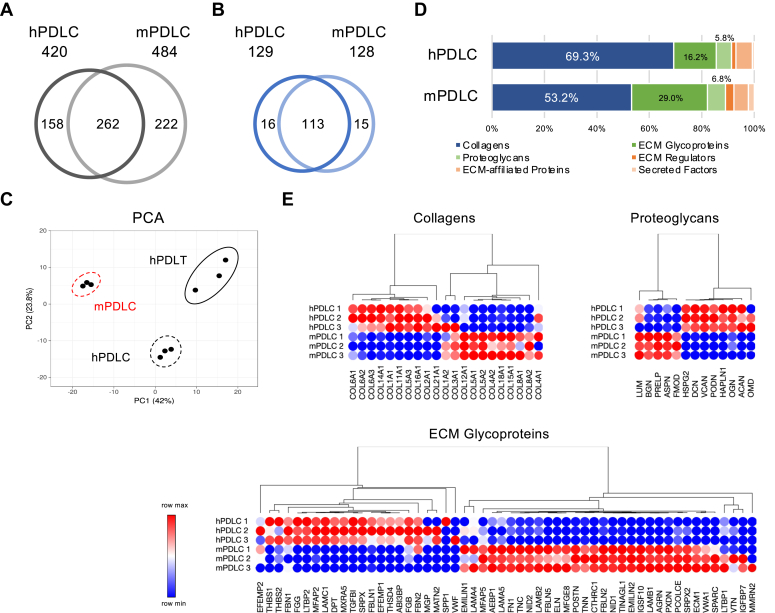
**ECM profiling of human periodontal ligament cells (hPDLC) and mouse periodontal ligament cells (mPDLC).** Venn diagram showing the number of detected proteins (*A*) and matrisome proteins (*B*) in hPDLC and mPDLC. *C*, PCA of the ECM profile on hPDLC, mPDLC, and hPDL tissue (hPDLT). *D*, Matrisome profile of hPDLC and mPDLC. *E*, Heatmap showing relative abundance patterns of core matrisome proteins in hPDLC and mPDLC.

Although the mouse model has generally been used for biological research because of its genetic similarity to humans and ease of acquisition and manipulation, it also serves as a valuable tool for elucidating disease etiology and developing potential therapeutic interventions. However, certain limitations must be considered when extrapolating findings from murine studies to human biology. Therefore, it is essential to consider species-specific differences in anatomy and physiology, as well as in ECM composition. In this study, we integrated a unified FragPipe/MSFragger + IonQuant workflow for consistent peptide identification and quantification with orthogene for precise 1:1 ortholog mapping, thereby facilitating robust, label-free comparative proteomics between mice and humans. The number of detected proteins was higher in mouse PDLCs than in human PDLCs, although counts were comparable at the matrisome protein level (Fig. 7, *A* and *B*). PCA demonstrated that the ECM compositions of mouse PDLCs, human PDLCs, and human PDL tissue were distinct from one another (Fig. 7*C*). The matrisome profile further revealed that human PDLCs contained a higher proportion of collagens, whereas mouse PDLCs were enriched in ECM glycoproteins (Fig. 7*D*). The heatmap showed that protein abundance patterns differed markedly between human and mouse PDLCs at the individual protein level (Fig. 7*E*).

## Discussion

The ECM is a complex and dynamic network of macromolecules consisting of a diverse array of proteins; these ECM proteins are covalently cross-linked to form a rigid tissue architecture, thereby making it challenging to accurately determine the protein profile. Although technical limitations still exist, substantial efforts have been made to address these challenges (56). The solubilization efficiency of proteins significantly influences their composition in mass spectrometry-based proteomics. Therefore, in this study, we first evaluated the impact of different protein extraction methods on ECM composition. We demonstrated that conventional chaotropic extraction methods, such as the use of UA/SDS and Gnd-HCl alone, are insufficient to elucidate the complete ECM profile. The fractions prepared from the insoluble remnants after Gnd-HCl extraction (Fr 3/3+) were highly enriched in type I collagen, indicating that a significant portion of the structural ECM components are overlooked by conventional protein extraction methods. The findings suggest that prior research investigating PDL tissue or PDLC ECM composition through the use of the UA/SDS extraction method (32, 49, 50) may have inaccurately quantified ECM composition, potentially underrepresenting collagen content. Furthermore, variations in protein quantification algorithms, such as spectral-counting versus intensity-based quantification, may influence the results. Through a series of comparative analyses, we established that one-step HA/Gnd-HCl extraction with deglycosylation (Fr 4+) provided a robust ECM profile.

HA-Fr (Fr 3/3+) was obtained from the insoluble remnants of Gnd-HCl extracted via chemical digestion with HA. This fraction contains a significantly higher proportion of fibrillar collagens (e.g., types I and III) and certain ECM glycoproteins (e.g., FN1, FBN1, and FBN2). These poorly soluble ECM, primarily composed of extensively cross-linked collagens and microfibrils, remained undetected when only conventional extraction buffers were employed. Consistent with this, a previous study identified a high abundance of fibrillar collagen in the insoluble remnants of Gnd-HCl extracted from various tissue types: >90% in bone, 75% in skin, and 52% in lungs (22). Despite the increasing solubilization of the ECM by HA, the complexity of its cleavage rule needs to be considered. Although HA primarily targets NG sites (57), its activity extends to other Asn-X (NX) sites, where X is any other amino acid except cysteine (22). During the database search, these nonspecific NX cleavages and the oxidation of some amino acid residues induced by the chemical digestion of HA need to be considered.

Our data demonstrated that deglycosylation by PNGase F influenced the number of identified matrisome proteins; however, it did not substantially affect the overall matrisome protein proportions. Previous studies have shown that deglycosylation by PNGase F significantly enhances the identification of proteoglycans but does not substantially affect the identification of collagen or ECM glycoproteins (17). Moreover, other glycosaminoglycan-digesting enzymes, such as chondroitinase ABC and heparinase II can further improve proteoglycan coverage (13). However, in this study, proteoglycans besides HSPG2 were successfully detected using conventional methods, such as UA/SDS and Gnd-HCl extraction, even without deglycosylation. Our data indicate that deglycosylation by PNGase F may be useful for the identification of low-abundance matrisome proteins, especially ECM glycoproteins and ECM regulators. Notably, the impact of deglycosylation cannot be solely attributed to the cleavage of N-glycans, as a low correlation was observed between the number of N-glycosylation sites and the effect of deglycosylation on protein proportion. One possible explanation is an alteration in the protein-binding affinity of glycoproteins. As glycosylation plays an important role in ECM protein stability (58), the removal of N-glycans could affect the binding affinity between certain combinations of the protein-protein interactions, which affects protein identification efficiency. While the increased complexity and additional expenses associated with the deglycosylation procedure needs to be considered. Consequently, the necessity of deglycosylation should be carefully evaluated based on specific research objectives.

Post-translational modifications (PTMs) are crucial to ECM biology, as they enhance protein chemistry and refine ECM behavior. The hydroxylation and subsequent glycosylation of proline and lysine residues stabilize the collagen triple helix and facilitate orderly fibril assembly (59, 60). Enzymatic cross-linking, primarily mediated by lysyl-oxidase family enzymes, connects adjacent collagen molecules, forming the three-dimensional scaffold that dictates tissue mechanics (61). Phosphorylation introduces an additional regulatory dimension: phosphate groups on osteopontin, dentin matrix protein-1, laminins, and other ECM components modulate mineral deposition, matrix organization, and cell-ECM signaling (62). Given that these PTMs vary with development, remodeling, and disease, systematically mapping them can provide deeper insights into ECM function and pathology. In our workflow, we account for key collagen PTMs by identifying hydroxylation of proline (Hyp) and lysine (Hyl), ensuring accurate identification and quantification of heavily modified ECM proteins (63). To this end, we expanded our analysis of PTMs, specifically focusing on the Hyp and Hyl on type I collagen (COL1A1 and COL1A2), as these modifications are crucial for triple-helix stability and for subsequent intermolecular crosslinking that defines tissue mechanics. We successfully detected Hyp and Hyl residues in COL1A1 and COL1A2 (Supplementary Fig. S1; a comprehensive list of PTMs detected in this study is presented in Supplementary Table S1). Site-resolved analysis revealed a general tendency for native tissue (hPDLT) to exhibit higher hydroxylation levels compared with cell-derived ECM (hPDLC); however, limited peptide coverage and reproducibility prevented statistical confirmation of this trend, and several expected residues were not consistently detected, due to the inherent stochasticity of DDA proteomics. Notably, the canonical cross-linking lysine sites (e.g., COL1A1 K87, K930; COL1A2 K87, K933) were not reliably observed. This reflects a general limitation of DDA workflows in capturing low-abundance or structurally constrained PTMs, as has been demonstrated in comparative analyses (64). To overcome these limitations, more targeted strategies, such as the stable isotope-labeled collagen approach (65), are likely more suitable for future work focused on biologically critical cross-linking sites (60). Future integration of dedicated PTMs with chemical digestion-assisted ECM proteomics will offer the broader, systems-level perspective necessary for a comprehensive understanding of ECM regulation in health and disease.

ECM proteins are often present in lower abundance than intracellular proteins, resulting in cellular proteins dominating the proteome. Therefore, decellularization has been used to enrich matrisome proteins. However, this procedure significantly reduces the number of matrisome-associated proteins (66). Among the various decellularization methods and reagents available (15, 67), we employed RIPA buffer consisting of a detergent cocktail (SDS, sodium deoxycholate, and NP-40). Our data revealed that even after decellularization with RIPA buffer, a considerable amount of cellular proteins, particularly cytoskeletal proteins, remained. In contrast, decellularization inevitably results in the loss of the easily soluble ECM components to some extent (22). These adverse effects underscore the importance of carefully selecting and optimizing decellularization methods to minimize ECM protein loss. Consequently, we employed the sequential removal of cellular proteins, initially through physical decellularization using RIPA buffer, followed by in silico decellularization using a Matrisome database. This approach minimizes the loss of ECM proteins, thereby preserving the native ECM composition.

This is the first study to elucidate the reliable ECM composition of human PDL tissues. Data demonstrated that collagen constitutes over 80% of the ECM in human PDL tissue, with type I collagen being predominant, followed by types III, VI, and XI. Moreover, the proportion of type I collagen was higher (∼80% of total collagen) than that previously reported (75%) (68, 69). Among the non-collagenous proteins, POSTN, DPT, LUM, DCN, and ASPN were the most abundant, highlighting their potential importance in PDL function (70, 71, 72). Although previous studies, including ours, have sought to elucidate the ECM composition of the PDL under various conditions (30, 31, 32, 50), the proportion of large and heavily cross-linked proteins, particularly collagen, may have been underrepresented, because conventional protein extraction protocols were utilized in these studies. In this study, one-step chemical digestion achieved apparently complete solubilization of PDL tissue and PDLC-derived ECM; however, differential extraction efficiencies across matrices may bias quantitative recovery. Moreover, recent advances in LFQ proteomic workflows have enabled more reliable quantification and inter-sample comparison (41, 42, 43, 73).

Owing to the comprehensive and unbiased nature of our proteomic approach, we identified DPT as the second most abundant non-collagenous ECM protein in human PDL tissue. Interestingly, *Dpt*-knockout mice exhibit increased dermal elasticity, reduced dermal thickness, approximately 40% lower total collagen content, and highly irregular collagen fibril morphology (74). Furthermore, a recent *in vitro* study reported that DPT expression increases during the osteogenic differentiation of human PDLCs, and recombinant DPT significantly enhanced osteogenic marker expression and mineral deposition in human PDLCs (75). Further studies are warranted to clarify the role of DPT in the maintenance and regeneration of the PDL.

Despite the clinical significance of PDL in masticatory function, regeneration of PDL still impractical due to the complexities involved in cell differentiation kinetics. One promising approach is the use of PDLC sheets, which comprise PDLCs and their self-secreted ECM (55). The PDLC-derived ECM provides an appropriate environment for cells, which at least partially represents the *in vivo* conditions of PDL. In fact, in this study, we successfully determined the precise composition of the PDLC-derived ECM, showing that it partially mimics that of human PDL tissue, with a lower proportion of collagen. Enrichment analysis indicated that ECM-receptor interactions and cell-cell interactions are enhanced in the culture condition compared with that in their native tissue environment, which likely reflects adaptations to the *in vitro* environment and may have implications for the advantages in tissue engineering applications. Furthermore, cell culture is an essential tool for analyzing biological systems in controlled environments. Consequently, understanding the distinctions between *in vitro* and *in vivo* ECM statuses is of paramount importance. Moreover, it is possible to manipulate the ECM of cultured PDLCs to more closely resemble the PDL tissue by modifying the culture conditions. Thus, the ECM profile obtained in this study will serve as a foundation for the quality control and manipulation of components that could facilitate the improvement of cell-derived ECM. Although further investigation is necessary to validate our findings, the in-depth characterization of the PDLC-derived ECM presented herein provides valuable insights into the development of tissue-specific biomimetic ECM scaffolds for periodontal tissue regeneration.

In conclusion, this study demonstrates the efficacy of chemical digestion-assisted proteomics for comprehensively characterizing the ECM, particularly in tissues rich in cross-linked collagens, such as PDL. By optimizing chemical digestion and deglycosylation protocols, we achieved more accurate and robust quantification of ECM components compared to conventional extraction methods. Using this approach, we provide the first detailed ECM profile of the human PDL, highlighting its high collagen content and essential non-collagenous ECM proteins. Additionally, we elucidated significant changes in ECM composition when PDLCs were cultured *in vitro*, offering insights relevant to tissue engineering applications. This robust analytical workflow enables detailed ECM characterization across various tissues and conditions, enhancing our understanding of ECM biology. These findings inform innovative strategies for periodontal tissue regeneration and broader applications in regenerative medicine.

## Data Availability

The mass spectrometry proteomics data have been deposited to the ProteomeXchange Consortium via the jPOST repository (76) with the dataset identifiers PXD060346/JPST003540 for mouse PDLCs and JPST003579/PXD060345 for human PDL tissue and PDLCs. The complete lists of peptides and proteins detected in this study are provided in Supplementary Tables S2 and S3.

## Supplemental data

This article contains supplemental data (60, 64, 65).

## Conflict of Interest

The authors declare that they do not have any conflicts of interest with the content of this article.

## Supplementry Data

Supplementry Table S1

Supplementry Table S2

Supplementry Table S3

Supplemental Figure S1

Clinical-Checklist
