# Supplementary material for: Chemical Digestion-Assisted Proteomics Reveals the Extracellular Matrix Profile of Human Periodontal Ligament and its Alterations in Cultured Cell-Derived Extracellular Matrix
Source: Mol Cell Proteomics. 2025 Nov 10;24(12):101460. doi: 10.1016/j.mcpro.2025.101460 (PMC12753232; doi:10.1016/j.mcpro.2025.101460)
Supplement: Supplemental Methods [file mmc4.docx]

**Supplementary Methods: Hydroxylation of Proline and Lysine Residues in COL1A1 and COL1A2**

LFQ intensities were normalized at the replicate level using the total type I collagen signal as an internal reference. For each replicate, the summed intensity of COL1A1 and COL1A2 peptides (both modified and unmodified) was calculated and used as a scaling factor. All peptide intensities were divided by this factor to yield collagen-aware normalized values. Normalized peptide intensities were then aggregated to the level of individual hydroxylation sites (Hyp or Hyl) by summing the signals of all peptides covering the same residue. Group averages were calculated separately for hPDLC and hPDLT and t used for site-resolved LFQ mapping.

**Supplementary Results/Discussion:**

Both Hyp and Hyl residues of COL1A1 and COL1A2 were successfully detected (Supplementary Fig. 1; a comprehensive list of detected PTMs is presented in Supplementary Table S1). Our site-resolved analysis furthermore indicated a general tendency for native tissue (hPDLT) to exhibit greater hydroxylation than that in cell-derived ECM (hPDLC); however, limited peptide coverage and reproducibility, owing to the inherent stochasticity of DDA proteomics, has prevented statistical confirmation of this trend; additionally, several expected residues were not consistently detected. Notably, several canonical cross-linking lysine sites (e.g., COL1A1 K87, K930; COL1A2 K87, K933) were not reliably observed. This limitation reflects a general limitation of DDA workflows in capturing low-abundance or structurally constrained PTMs, as has been demonstrated in comparative analyses (1). To overcome these limitations, more targeted strategies, such as the stable isotope-labeled collagen approach (2), will likely be better suited for future studies of biologically critical cross-linking sites (3). Future integration of dedicated PTMs with chemical digestion-assisted ECM proteomics will offer the broader, systems-level perspective necessary for a comprehensive understanding of ECM regulation in health and disease.

**References**

1. Barkovits, K., Pacharra, S., Pfeiffer, K., Steinbach, S., Eisenacher, M., Marcus, K., et al. (2020) Reproducibility, Specificity and Accuracy of Relative Quantification Using Spectral Library-based Data-independent Acquisition. *Mol Cell Proteomics* 19, 181-197

2. Taga, Y., Kusubata, M., Ogawa-Goto, K., and Hattori, S. (2014) Stable isotope-labeled collagen: a novel and versatile tool for quantitative collagen analyses using mass spectrometry. *J Proteome Res* 13, 3671-3678

3. Yamauchi, M., Terajima, M., and Shiiba, M. (2019) Lysine Hydroxylation and Cross-Linking of Collagen. *Methods Mol Biol* 1934, 309-324
