# Supplementary figures and images for: Chemical Digestion-Assisted Proteomics Reveals the Extracellular Matrix Profile of Human Periodontal Ligament and its Alterations in Cultured Cell-Derived Extracellular Matrix
Source: Mol Cell Proteomics. 2025 Nov 10;24(12):101460. doi: 10.1016/j.mcpro.2025.101460 (PMC12753232; doi:10.1016/j.mcpro.2025.101460)

# Thant *et al.* Supplemental Fig. 1

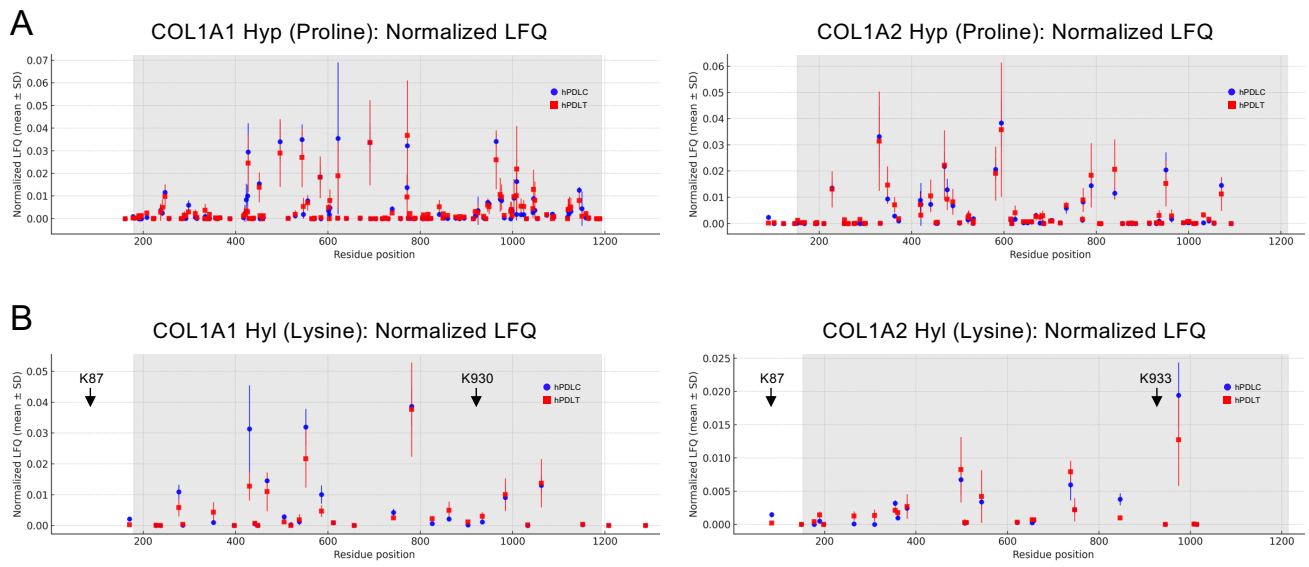

Supplement: Supplemental Figure S1 [file mmc5.pdf]
